# Improved Glycemic Control and Variability: Application of Healthy Ingredients in Asian Staples

**DOI:** 10.3390/nu13093102

**Published:** 2021-09-03

**Authors:** Stefan Gerardus Camps, Bhupinder Kaur, Joseph Lim, Yi Ting Loo, Eunice Pang, Terence Ng, Christiani Jeyakumar Henry

**Affiliations:** 1 Clinical Nutrition Research Centre (CNRC), Singapore Institute of Food and Biotechnology Innovation (SIFBI), Agency for Science, Technology and Research (A*STAR), Centre for Translational Medicine, 14 Medical Drive #07-02, MD 6 Building, Yong Loo Lin School of Medicine, Singapore 117599, Singapore; bhupinder_kaur@sifbi.a-star.edu.sg (B.K.); joseph_lim@sifbi.a-star.edu.sg (J.L.); yiting_loo@sifbi.a-star.edu.sg (Y.T.L.); 2 Health Promotion Board, 3 Second Hospital Avenue, Singapore 168937, Singapore; Eunice_Pang@hpb.gov.sg (E.P.); Terence_NG@hpb.gov.sg (T.N.); 3 Department of Biochemistry, Yong Loo Lin School of Medicine, National University of Singapore, Singapore 117543, Singapore

**Keywords:** glycemic variability, glycemic load, glycemic index carbohydrate quality, β-glucan, isomaltulose, chrononutrition

## Abstract

A reduction in carbohydrate intake and low-carbohydrate diets are often advocated to prevent and manage diabetes. However, limiting or eliminating carbohydrates may not be a long-term sustainable and maintainable approach for everyone. Alternatively, diet strategies to modulate glycemia can focus on the glycemic index (GI) of foods and glycemic load (GL) of meals. To assess the effect of a reduction in glycemic load of a 24 h diet by incorporating innovative functional ingredients (β-glucan, isomaltulose) and alternative low GI Asian staples (noodles, rice)on glycemic control and variability, twelve Chinese men (Age: 27.0 ± 5.1 years; BMI:21.6 ± 1.8kg/m^2^) followed two isocaloric, typically Asian, 24h diets with either a reduced glycemic load (LGL) or high glycemic load (HGL) in a randomized, single-blind, controlled, cross-over design. Test meals included breakfast, lunch, snack and dinner and the daily GL was reduced by 37% in the LGL diet. Continuous glucose monitoring provided 24 h glycemic excursion and variability parameters: incremental area under the curve (iAUC), max glucose concentration (Max), max glucose range, glucose standard deviation (SD), and mean amplitude of glycemic excursion (MAGE), time in range (TIR). Over 24h, the LGL diet resulted in a decrease in glucose Max (8.12 vs. 6.90 mmol/L; *p* = 0.0024), glucose range (3.78 vs. 2.21 mmol/L; *p* = 0.0005), glucose SD (0.78 vs. 0.43 mmol/L; *p* = 0.0002), mean amplitude of glycemic excursion (2.109 vs. 1.008; *p* < 0.0001), and increase in 4.5–6.5mmol/L TIR (82.2 vs. 94.6%; *p* = 0.009), compared to the HGL diet. The glucose iAUC, MAX, range and SD improved during the 2 h post-prandial window of each LGL meal, and this effect was more pronounced later in the day. The current results validate the dietary strategy of incorporating innovative functional ingredients (β-glucan, isomaltulose) and replacing Asian staples with alternative low GI carbohydrate sources to reduce daily glycemic load to improve glycemic control and variability as a viable alternative to the reduction in carbohydrate intake alone. These observations provide substantial public health support to encourage the consumption of staples of low GI/GL to reduce glucose levels and glycemic variability. Furthermore, there is growing evidence that the role of chrononutrition, as reported in this paper, requires further examination and should be considered as an important addition to the understanding of glucose homeostasis variation throughout the day.

## 1. Introduction

The prevalence of type 2 diabetes and related risk factors have increased dramatically across Asia in recent decades and Singapore is no exception. Improved economic prosperity, advances in technology, increased food production, availability and safety have allowed a shift towards a lifestyle with reduced physical activity and a diet with a greater overall carbohydrate intake, lower fiber and higher sugar and fat content [1]. In addition, the Asian phenotype, marked by relatively greater body fat within the normal body weight and BMI range, has been shown to be more susceptible to diabetes than the Caucasian phenotype [2]. Diabetes is often diagnosed at a lower body mass index (BMI) and a younger age and the transition from prediabetes to diabetes is more dramatic in Asians [3]. 

Chronic high blood glucose levels are considered a major risk factor for the onset of diabetes; in addition, greater glucose variability has independently been linked to the increased risk for the development of diabetes and cardiovascular diseases (CVD) [4,5,6], likely via a combination of increased inflammation, oxidative stress and endothelial dysfunction [7,8,9,10]. Glycemic variability (GV) refers to the fluctuations of glucose throughout the day as influenced by meals and the circadian rhythm. Fasting blood glucose (FBG) and glycosylated hemoglobin (HbA1c) are easy–to-perform standard reference measurements used in a clinical setting as an indication of glycemic control and for diabetes diagnosis [6]. FBG is measured after an overnight fast and corresponds closely to the lowest daily glucose values. It does not capture GV and does not reflect any post-prandial glucose levels that could be indicative of impaired glucose tolerance or pancreatic health. HbA1c is a robust biomarker for average blood glucose levels over a 3-month period and as such includes glucose peaks and valleys, though it does not reflect their extend and the level of GV [11]. Furthermore, the relationship between glucose and HbA1c may be influenced by ethnicity, smoking, glycation rates and conditions that affect the red blood cells [11]. At present, technological advancements have resulted in the increased application of continuous glucose monitoring (CGM) systems in diabetes care. CGM offers near real-time measurements of glucose levels and provides the ability to assess GV in detail which can be used to complement FBG and HbA1c in a clinical setting [6,12,13,14].

Diet strategies to manage glycemia have included frequent small meals [15] and reduction in carbohydrate content of diets [16]. However, limiting or eliminating carbohydrates may not be a long-term sustainable and maintainable approach for everyone. Alternatively, diet strategies to manage glycemia can focus on the glycemic index (GI) of foods and glycemic load (GL) of meals [17,18,19]. The glycemic index (GI) was first introduced in 1981 as a classification of the blood glucose raising potential of a 50g carbohydrate portion of foods and can be seen as a measure for carbohydrate quality [20]. Glycemic load (GL) combines both the quantity and quality of carbohydrates. There is substantial evidence to suggest that the consumption of low GI foods and a reduction in GL of a diet can result in a lower glycemic response and minimize blood glucose fluctuations which can reduce the risk of type II diabetes and cardiovascular diseases [19,21,22,23]. 

As part of dietary strategies, several functional food ingredients have been shown to moderate the postprandial glycemic response. Dietary fibers refer to plant components that are predominantly non-digestible by the human digestive system. β-glucan, a soluble fiber, which is a glucose polymer, can reduce glucose availability and its inclusion in foods can lower the postprandial glycemic response and the GI [24,25,26,27]. In addition, fiber can be a fermentable carbohydrate source for bacteria in the large intestine which can produce short chain fatty acids that may attenuate the subsequent or later glycemic excursion [28,29,30]. Isomaltulose is a disaccharide composed of glucose and fructose just like sucrose, though with a α-1,6-glycosidic bond instead of α-1,2-glycosidic bond. The α-1,6-glycosidic bond is hydrolised more slowly by the sucrase–isomaltase complex located in the wall of the small intestinal cells. As a result, the rate of absorption of isomaltulose is relatively slow; nonetheless, the glucose and fructose are fully absorbed [31,32]. Previous studies have shown that isomaltulose can improve glycemic responses in individuals with and without diabetes [32,33]. 

To enhance the adoption of healthier foods of low GI by consumers and the food industry, it is necessary to develop and offer these foods based on staples consumed in Asian countries. The current study used alternative low GI Asian staples and healthier food prototypes and were specifically formulated based on Singapore’s Healthier Choice nutrient guidelines and included in this study for physiological validation [34]. In this study, the effect of a reduction in glycemic load of 24h diet by incorporating innovative functional ingredients (β-glucan, isomaltulose) and alternative low GI Asian staples (noodles, rice) on glycemic control and variability in healthy Chinese adults was examined.

## 2. Materials and Methods 

### 2.1. Subjects

Fifteen healthy Chinese male subjects between 21 and 40 years were recruited by a variety of methods including flyers, online advertisement and the research centre’s volunteer database. Volunteers had a body mass index (BMI) between 18.5 to 25 kg/m^2^, had fasting blood glucose below 6 mmol/L and had a blood pressure below 140/90 mm·Hg. Exclusion criteria were metabolic diseases (such as diabetes, stage II hypertension etc.), known glucose-6-phosphate dehydrogenase deficiency (G6PD deficiency), medical conditions and/or taking medications known to affect glycemia (glucocorticoids, thyroid hormones, thiazide diuretics), intolerances or allergies to test foods, sports participation at a competitive and/or endurance levels, intentional food intake restriction and smoking. One drop-out and two incomplete data sets resulted in a total of 12 included data sets for analysis.

The study was conducted at the Clinical Nutrition Research Centre (CNRC), Singapore, in accordance with the guidelines laid down in the Declaration of Helsinki. All procedures involving human participants were approved by the Domain Specific Review Board (DSRB) of the National Healthcare Group, Singapore (Reference no. 2017/00994). The protocol was well explained to the volunteers and written informed consent was obtained from each subject before participation. This study was registered in the clinicaltrial.gov registry as NCT03703544.

### 2.2. Study Design

In a randomized, single-blind, controlled, cross-over design, subjects attended two test sessions separated by a wash-out period of at least 3 days. The two test sessions included either a reduced glycemic load (LGL) or a high glycemic load (HGL) 24 h meal sequence (breakfast, lunch, snack and dinner). The 24 h meal sequence was based on typical Asian foods and included innovative functional ingredients (β-glucan, isomaltulose) and alternative low GI Asian staples (noodles, rice). The macronutrient and energy composition of the meals is provided in Table 1 and the ingredients of the meals in Table 2. Participants were advised to not perform any rigorous activities three days prior to the study and during the study session. Each test session spanned over three consecutive days from around 16:00 on Day 1 until 9:00 on Day 3 consisting of at least 24 h continuous glucose monitoring (CGM). On Day 2, participants arrived at the centre around 08:30 to 09:00 following a 10–12 h overnight fast. Participants consumed the LGL or HGL breakfast and lunch at the centre and were provided with the LGL and HGL snack and dinner to bring home. Online computer software was used for simple randomization of the sequence of the treatment diets (http://www.randomizer.org/ (accessed on 9 August 2021)) [35].

### 2.3. Treatment Meals

The two test sessions included either a reduced glycemic load (LGL) or a high glycemic load (HGL) 24 h meal sequence (breakfast, lunch, snack and dinner). The two diets had an equal amount of available carbohydrates and were closely matched in energy content and macronutrient distribution (Table 1). The glycemic load was determined for each meal and cumulative for each diet. The 24 h meal sequence was based on typical Asian foods and included innovative functional ingredients (β-glucan, isomaltulose) and alternative low GI Asian staples (noodles, rice) (Table 2). The β-glucan was incorporated into the noodles at 4.2% and Isomaltulose was administered as a sucrose substitute in a jelly and tea. 

### 2.4. Interstitial Glucose Measurement

Continuous glucose monitoring (CGM) (iPro™2 Professional CGM-Medtronic MiniMed, Northbridge, CA, USA) was used to measure glycemic response, defined as the primary outcome. The insertion was performed on Day 1 at approximately 16:00 and the sensor was removed on Day 3 of the study at 09:00. Data was collated and processed using online software (Medtronic Diabetes CareLink iPro; carelink.minimed.eu). The data reported in this paper represent 24 h interstitial glucose readings recorded every 5 min from the start of breakfast on Day 2 at approximately 09:00 until 24 h later at approximately 09:00 on Day 3. During each test session, the CGM sensor was calibrated against finger-stick blood glucose measurements four times a day before every meal and before sleeping using the FreeStyle Optium Neo Blood Glucose meter (Abbott Laboratories). A cross-over design with a minimum of 8 subjects would be sufficient to detect a 15% change in area under the glucose curve (24 h) with a power of 0.85 at a significance level of 0.05 [36].

### 2.5. Statistics

All statistical analyses were performed using Statistical Package for the Social Sciences version 23 (IBM Corp. Armonk, NY, USA). Values are presented as mean ± SEM unless otherwise stated. Prior to statistical analysis, the normality of the data was assured using the Shapiro-Wilks test. Statistical significance was set at *p* < 0.05.

The primary outcome was to determine how a 24 h meal sequence based on a LGL diet using functional ingredients and healthier alternative food staples impacts the 24h glycemic response and variability. Firstly, the baseline glucose value for each subject was determined from the 1h average CGM interstitial glucose readings in a fasted state from 06:00 to 07:00 on day 2 and used to calculate the change in glucose level for the 24 h. Glycemic response was expressed as the incremental area under the curve (iAUC), calculated using the trapezoidal approximation method and the change in glucose above baseline (baseline-corrected) [37]. The total area under the curve (tAUC) was calculated using the trapezoidal approximation method and included all the area under the curve using the absolute value of glucose [37]. Secondly, the following glycemic parameters related to glycemic variability were determined for each subject: highest glucose concentration (Max), max glucose range (difference highest glucose value—lowest glucose value), glucose standard deviation (SD), time in range (TIR), and mean amplitude of glycemic excursion (MAGE). TIR and MAGE were only assessed as 24 h indicators for GV [38,39]. The TIR for individuals with diabetes is defined as the time spent within the target glucose range of 3.9–10.0 mmol/L [13,14]. For the current study, the cut-off points were adjusted to 4.5–6.5 mmol/L to increase the detection power of possible difference between the treatments in healthy subjects. These cut-off values were based on the 10th and 90th percentile values for all the 5-min data points of the 24 h CGM data (both diets). MAGE was calculated using the extensively reviewed EasyGV software (available for free at www.easygv.co.uk, accessed on 9 August 2021) [40]. All parameters were determined for the full 24 h period and additionally, glucose iAUC, Max, range and SD were determined for each meal-specific 2 h post-prandial period.

## 3. Results

Anthropometric measurements and characteristics of the subjects can be found in Table 3.

The cumulative glycemic load of the LGL was 37% lower compared to the HGL diet (165 vs. 263) and the reduction was fairly consistent among the four meals: −31% for breakfast, −43% for lunch, −36% for snack, and −34% for dinner (Table 1). This equated to a 38% reduction in GL/1000 kcal for the LGL compared to HGL diet (77 vs. 124 GL/1000kcal) and the following reduction per meal: −33% for breakfast, −45% for lunch, −36% for snack, and −33% for dinner.

**Figure 1:**
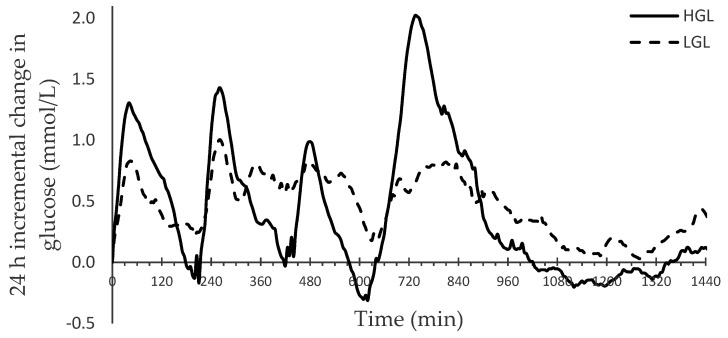
Change in 24 h glycemic response over time for the LGL and HGL diet. LGL: reduced GL; HGL: high GL.

The incremental change in glucose compared to baseline is depicted in Figure 1. The major glycemic parameters of the 24 h CGM assessment are shown in Table 4. There was no difference in mean glucose (5.69 ± 0.08 vs. 5.71 ±0.11 mmol/L), total glycemic response (8097 ± 383 vs. 8078 ± 547 mmol/L) and incremental area under the curve (732 ± 96 vs. 870 ± 163 mmol/L; *p* = 0.448) over 24 h between the LGL and HGL diet. Parameters indicative of glycemic variability showed that over 24 h, the LGL diet resulted in a decrease in: glucose Max (−15%; *p* = 0.0024), glucose range (−42%; *p* = 0.0005), glucose SD (−45%; *p* = 0.0002), mean amplitude of glycemic excursion (−52%; *p* < 0.0001), and increase in 4.5–6.5mmol/L TIR (+15% *p* = 0.009), compared to the HGL diet. In summary, the current data suggests a great improvement in GV over 24h following the LGL diet.

Meal-specific analysis revealed an improvement in almost all determined glycemic parameters for each of the meals (Table 4). The greatest improvements were observed after the LGL dinner: iAUC (−76%; *p* = 0.0015), glucose Max (−16%; *p* = 0.0084), glucose range (−49%; *p* = 0.0124), glucose SD (−55%; *p* = 0.0076) compared to the HGL dinner. Comparing the improvements per meal relative to their reduction in GL (improvement factor = % reduction in iAUC/% reduction in GL/1000kcal) between the meals showed an IF of 1.27 for breakfast, 1.27 for lunch, 1.62 for the snack and 2.28 for the dinner respectively. In other words, each percentage point reduction in GL during dinner, reduced the iAUC with 2.28% compared to only 1.27% during breakfast and lunch.

## 4. Discussion

The 24 h reduced GL diet resulted in a much improved 24 h glycemic response compared to the control high GL diet as indicated by: reduced glycemic variability, lower peak glucose levels, greater time in range, and improved post-prandial glycemic response after each meal. The “healthier choice” diet with innovative ingredients improved glycemic variability and glycemic control, two major independent risk factors in the development and progression of type 2 diabetes. Therefore, this study validates the use of low GI foods to reduce GL as an alternative dietary strategy to limiting carbohydrate intake alone in Asians to manage glycemia. The reduction in GL was accomplished by incorporating innovative functional ingredients (β-glucan, isomaltulose) and replacing Asian staples with alternative low GI carbohydrate sources to improve carbohydrate quality. Notably, a relatively greater beneficial effect on glucose control was observed during the evening compared to the morning.

Consistently, studies have demonstrated that oat and barley products which are natural sources of β-glucan and starchy products fortified with β-glucan aid in reducing glycemic response without an increase in insulin secretion to mediate increased glucose clearance [41]. Likely mechanisms underlying these observations are an increased viscosity of chymus, a decreased carbohydrate digestion rate, and a reduced enzyme-substrate interaction [42,43]. Fortification with β-glucan has been a successful method to modify the digestibility of starchy foods and has been applied in various starch product such as spaghettis and Indian flat bread [43,44]. In the current study, the addition of β-glucan fiber to wheat flour noodles reduced the glycemic response, as reflected by a decrease in glucose peak, a reduced glucose range and a reduced glucose variability compared to identical noodles without β-glucan. In line with previous studies, this demonstrates the diverse application of β-glucan and its consistent beneficial effect in real food products [43,44,45].

Isomaltulose has a low GI and studies have repeatedly shown a decreased glycemic response compared to sucrose without an increase in insulin as the underlying mechanism [32,46,47]. Human enzymes studies have demonstrated that, for glucose-fructose disaccharides, the digestion rate of isomaltulose is much slower compared to sucrose, underpinned by the slower hydrolysis by the sucrose-isomaltase complex [48]. Other studies observed that greater amounts of glucose were retained by the liver, [47] as less glucose was released into the systemic circulation, resulting in a reduced glycemic response following isomaltulose consumption [47]. Our results showed that the inclusion of isomaltulose as part of a 24 h LGL diet can aide in the decrease of 24 h glycemic variability and can help to decrease the glycemic response, peak and range after a meal. These results are in line with previous studies on isomaltulose inclusion that found an acute attenuation of the glycemic and insulinemic response [49,50] and a 24 h study that showed a moderation of the glycemic response and variability [51].

In the current study an overall greater relative reduction in post-prandial glycemic response was observed in the evening following a reduced GL diet. The results showed that consumption of the LGL versus HGL diet at dinner, resulted in a greater relative reduction in the glucose iAUC, maximum value, range and variability (SD and MAGE), compared to the decrease earlier on the day following the LGL breakfast and lunch. Our study was not specifically designed to assess the effect of mealtime on glucose response, as the meals for breakfast, lunch and dinner were not identical. Nonetheless, this important observation on mealtime effect may be of clinical relevance. In addition, the relative reduction in GL between the HGL and LGL meals was consistent. Furthermore, when the relative improvement in iAUC was adjusted for the relative decrease in GL for each meal, the results indicated that for each 1% reduction in GL, the iAUC was reduced with 2.28% percentage point during dinner compared to only 1.27% during breakfast and lunch.

Our findings are in line with Morgan et al. who found that a low GI diet lowered the post-prandial glucose response both in the morning and the evening, though to a greater extent in the evening [52]. Furthermore, Gibbs et al. showed a significantly reduced post-prandial glucose peak following a low GI meal compared to a high GI meal in the evening but not in the morning. On the other hand, their results indicated a greater improvement in iAUC following a low GI meal in the morning and concluded that low GI foods are of less value for glycemic control in the evening than the morning [53]. Haldar et al. found a similar relative reduction in glucose iAUC following a low versus high GI meal for breakfast and dinner [54]. A greater detrimental effect of the consumption of high carbohydrate content and high GI meals in the evening may partially be attributed to the circadian control of glucose metabolism. The central circadian clock in the hypothalamus (suprachiasmatic nucleus), the peripheral clocks as found in the pancreas, and endogenous circadian rhythms in hormones (cortisol, melatonin) reduce glucose tolerance in the course of the day. Morris et al. suggest that the circadian modulation of β-cell function in the pancreas reduces insulin sensitivity from morning to evening and decreases early-phase insulin response in the evening [55,56]. A reduction in pancreatic insulin function later in the day may be required for physiological rest, repair, regeneration and normal insulin secretion function [57]. The relationship between diet and circadian rhythm involves factors such as meal timings and nutrients, known as chrononutrition. Based on our findings, improving carbohydrate quality of the later meals of the day may be most beneficial for the best relative effect on daily glycemia. Along these lines, a modified macronutrient composition by increasing protein and fat content, may improve postprandial glycemia in the evening when glucose tolerance is lowest and should be investigated.

Our study further illustrates the limited use and application of single point measurements or average glucose values over a 24 h period [12,13,14]. The study eloquently describes how glycemic variability and time in range based on continuous glucose monitoring are important markers of metabolic homeostasis. Moreover, glycemic variability is an independent risk factor for the development of diabetes and cardiovascular diseases. In our study, between the LGL and HGL diet, 24 h glucose values were almost identical, and there was no significant difference in the 24 h iAUC values. This response hides a significant metabolic observation in relation to glucose excursions and variability. Figure 1, which shows the change in glucose values over time for both diets, reveals two distinct glucose responses not picked up by either of the previous parameters. On the other hand, over 24 h, the glucose standard deviation (−45%) and mean amplitude of glycemic excursion (−52%), both markers of glucose variability, were greatly reduced. Furthermore, glucose peak values (−15%), time in range (+15%) and time above range (−69%) analysis showed a significant improvement over 24 h following the reduced GL diet. Our data shows the benefit of a detailed glucose analysis and it is increasingly clear that the application of continuous glucose monitoring (CGM) systems in diabetes care and research provides more insights. CGM offers near real-time measurements of glucose levels and provides the ability to assess GV in detail [6,12,13,14]. Although this study was done in healthy subjects, the difference in glycemic variability markers and time in range show considerable difference between the low and high GL diet. We therefore speculate that in those with prediabetes and type 2 diabetes these parameters would be further attenuated and lead to improved metabolic health outcomes.

A minor limitation was the male-only study population, though similar results can be expected in women as previous studies have not demonstrated gender differences in mixed study populations [33,58]. The diets were closely matched for available carbohydrates and calories from fat and protein, though there may have been small differences in fat quality between the diets. Since the study outcomes focused on glycemia, we did not evaluate measures of fat quality like the ratio between saturated and unsaturated fat and its potential effect on glycemia [59,60]. Although the current study compared a full 24 h diet window, the findings should be validated over longer periods in future research.

## 5. Conclusions

The current results validated the glycemic load reduction strategy by replacing staples with “healthier choice” options containing innovative functional ingredients (β-glucan, isomaltulose) and replacing Asian staples with alternative low GI carbohydrate sources. Functional ingredients can improve the carbohydrate quality and the inclusion of such ingredients is a potential way to improve postprandial glycemia and glycemic variability. These observations provide substantial public health support to encourage the consumption of staples of greater carbohydrate quality as a viable alternative to the reduction in carbohydrate intake alone for glycemic control. Increased opportunities for research and development of highly palatable, low GI, fortified Asian food staples is required to aid in the prevention and treatment of diabetes among Asians. In addition, there is growing evidence that the role of chrononutrition requires further examination and should be addressed in the light of practical nutrition recommendations.

## Figures and Tables

**Table 1:** 24 h diet macronutrient and energy composition.

	Total Kcal (E% C/P/F)		aCHO (g)		Fiber (g)		GL	
	LGL	HGL	LGL	HGL	LGL	HGL	LGL	HGL
Breakfast	409 (51/15/34)	398 (52/13/35)	52	52	15.2	4.3	27	39
Lunch	583 (76/12/12)	561 (78/10/12)	111	109	3.2	1.8	54	95
Snack	361 (83/2/15)	361 (83/2/15)	75	75	0.9	0.9	30	47
Dinner	795 (64/12/24)	805 (63/13/23)	127	127	9.9	6.2	54	82
Total	2147 (68/11/21)	2125 (68/11/21)	365	363	29.2	13.2	165	263

E% C/P/F: energy percentage from available carbohydrate, protein and fat; aCHO: available carbohydrates GL: glycemic load; LGL: reduced GL; HGL: high GL.

**Table 2:** Foods used to construct the test meals provided in the study.

	LGL Ingredients	HGL Ingredients
Breakfast	*Yellow noodles (beta-glucan, 4.2%)*, spinach, minced pork, light and dark soya sauce, oil	*Yellow noodles (control)*, spinach, minced pork, light and dark soya sauce, oil
Lunch	*Parboiled basmati rice*, pork balls, sesame seeds, knorr seasoning, chamomile tea, *isomaltulose (25 g)*	*Glutinuous rice*, pork balls, sesame seeds, knorr seasoning, chamomile tea, *sucrose (25 g)*
Snack	Jelly, chamomile tea, *isomaltulose (55 g)*, oreo cookies	Jelly, chamomile tea, *sucrose (55 g)*, oreo cookies
Dinner	*Pad thai glass noodles with tofu*, wonton, chamomile tea, *isomaltulose (22 g),* oil, *rice biscuits*	*Teriyaki chicken with rice*, wonton, chamomile tea, *sucrose (22 g)*, oil, *potato chips*

Different ingredient indicated in italic font; LGL: reduced GL; HGL: high GL.

**Table 3:** Anthropometric and physiological parameters of subjects.

(*n* = 12)	Mean ± SD
Age (years)	27.0± 5.1
Height (m)	1.72 ± 0.04
Weight (kg)	63.8 ± 6.5
BMI (kg/m^2^)	21.6 ± 1.8
Body Fat (%)	16.2 ± 4.1
Systolic blood pressure (mm/Hg)	119.8 ± 8.4
Diastolic blood pressure (mm/Hg)	80.5 ± 6.1
Waist circumference (cm)	74.1 ± 4.2
Hip circumference (cm)	93.6 ± 4.4
Fasting blood glucose (mmol/L)	4.6 ± 0.7

BMI: Body Mass Index.

**Table 4:** Glycemic parameters for the LGL and HGL 24 h diet.

.	HGL		LGL		ΔHGL-LGL (%)	*p*-Value
	Mean	SEM	Mean	SEM		
**24 h average**	5.71	0.11	5.69	0.08	0%	0.806
**24 h iAUC**	869.8	163.1	731.7	95.5	−16%	0.448
**24 h Max**	8.12	0.33	6.90	0.10	−15%	0.002
**24 h ΔMax-Min**	3.78	0.34	2.21	0.13	−42%	0.000
**24 h SD**	0.779	0.079	0.429	0.041	−45%	0.000
**24 h MAGE**	2.109	0.170	1.008	0.073	−52%	0.000
**24 h TIR <4.5 mmol/L**	3.5%	1.2%	0.9%	0.5%	−73%	0.070
**24 h TIR 4.5–6.5 mmol/L**	82.2%	3.4%	94.6%	1.9%	15%	0.001
**24 h TIR >6.5 mmol/L**	14.4%	3.2%	4.4%	1.8%	−69%	0.001
**Breakfast iAUC**	117.1	25.8	68.6	12.3	−41%	0.117
**Breakfast Max**	6.88	0.24	6.23	0.15	−9%	0.020
**Breakfast ΔMax-Min**	2.04	0.27	1.20	0.08	−42%	0.008
**Breakfast SD**	0.571	0.067	0.289	0.083	−49%	0.008
**Lunch iAUC**	114.5	21.8	49.0	6.6	−57%	0.010
**Lunch Max**	7.00	0.27	6.45	0.14	−8%	0.047
**Lunch ΔMax-Min**	2.08	0.25	1.08	0.07	−48%	0.001
**Lunch SD**	0.591	0.077	0.317	0.020	−46%	0.003
**Snack iAUC**	65.6	13.3	27.2	7.7	−58%	0.022
**Snack Max**	6.73	0.26	6.48	0.12	−4%	0.286
**Snack ΔMax-Min**	2.07	0.25	1.25	0.11	−40%	0.007
**Snack SD**	0.603	0.074	0.340	0.037	−44%	0.001
**Dinner iAUC**	151.5	27.5	36.7	6.3	−76%	0.001
**Dinner Max**	7.85	0.38	6.58	0.14	−16%	0.004
**Dinner ΔMax-Min**	2.80	0.45	1.44	0.13	−49%	0.012
**Dinner SD**	0.883	0.163	0.397	0.039	−55%	0.008

LGL: reduced GL; HGL: high GL; iAUC: incremental area under the curve; SD: standard deviation; MAGE: mean amplitude of glycemic excursion; TIR: time in range.

## Data Availability

The data presented in this study can be requested from the corresponding author.
